# Kerion Celsi in a Nepalese Boy: An Underdiagnosed Cause of Scalp Swelling

**DOI:** 10.1155/2021/5527974

**Published:** 2021-06-24

**Authors:** Ranjana Parajuli, Ashish Lal Shrestha, Niranjan Nayak, Shishir Gokhale, Kundan Gautam, Shishir Subedi

**Affiliations:** ^1^Department of Microbiology, Grande International Hospital, Dhapasi, Tokha Road, Kathmandu, Nepal; ^2^Department of Pediatric Surgery, Grande International Hospital, Dhapasi, Tokha Road, Kathmandu, Nepal; ^3^Department of Medical Microbiology, Manipal College of Medical Sciences, Pokhara, Nepal

## Abstract

Tinea capitis (TC) is an infection of the scalp and hair caused by a dermatophyte fungus. Typically caused by the zoophilic and geophilic species of *Microsporum* and *Trichophyton*, it remains the commonest cutaneous fungal infection in children. A 9-year-old Nepalese boy was evaluated in outpatients for multiple boggy scalp lesions for two months. Suspecting a bacterial etiology, the lesions were excised and sent for cultures. While bacterial cultures failed to grow, endothrix spores were readily detected in potassium hydroxide preparation and histopathology. *Trichophyton tonsurans* was identiﬁed by the phenotype method and later conﬁrmed by matrix-assisted laser desorption ionization-time of ﬂight (MALDI-TOF). Systemic antifungal therapy for 6 weeks along with local wound dressings resulted in complete recovery. At 2-year follow-up, focal alopecia was seen; however, no recurrence was noted.

## 1. Introduction

Kerion is an inﬂammatory fungal infection affecting the hair follicles of the scalp, characterized by boggy swelling, purulent discharge, alopecia, and cervical lymphadenopathy. Although more commonly noted in children, often it is an infrequent and, at times, difficult to treat, a condition of the scalp [1]. Of the different pathogens implicated in its cause, the dermatophytes of genera *Trichophyton* and *Microsporum* are considered the predominant ones [2].

We report a case of kerion, which was initially thought to be a pyogenic swelling of bacterial origin based upon its clinical characters and treated with surgical excision. The diagnosis was obtained on evaluation for fungal pathogenesis. We, therefore, recommend a strong index of clinical suspicion supported by relevant microbiological analysis to avoid its misdiagnosis.

## 2. Case Illustration

**Figure 1:**
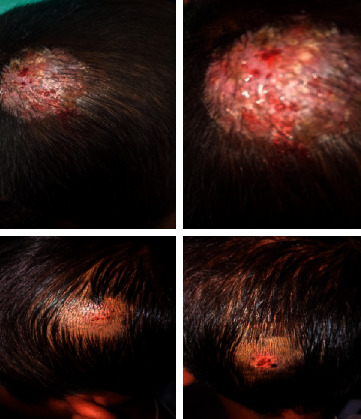
Boggy lesions over both the parietal scalp and occiput at presentation.

A previously healthy 9-year-old boy was evaluated for multiple painful scalp lesions for 2 months associated with focal alopecia. The lesions had started following a history of fall at the playground in the recent past. This was followed by the development of circular lesions that were itchy, to begin with. With failed attempts at treatment with oral antibiotics and topical antifungals, the lesions had increased in size evolving into boggy swellings with numerous pus points and black spots, as shown in Figure 1. The lesions were located over the parieto-occipital regions, the largest of which over the right parietal scalp measured 5 × 5 cm.

The hairs over the affected region were highly fragile, and the samples of hairs examined with 10% potassium hydroxide (KOH) revealed endothrix spores along the hair shaft.

**Figure 2:**
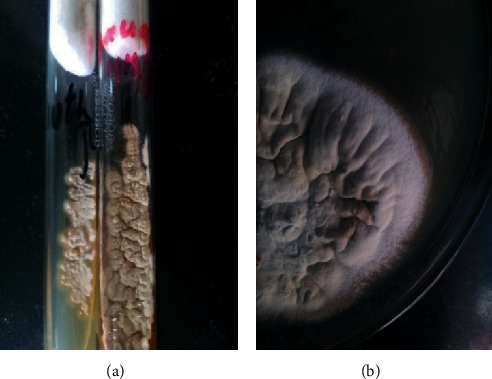
(a) DTM; (b) SDA with dermatophyte growth and features of *T. tonsurans*.

The excised lesions sent for bacterial culture were sterile. Fungal culture of the same on Sabouraud dextrose agar (SDA) grew small yellowish starry colonies on the 5^th^ day. On further incubation, the whitish colony with suede-like to powdery surface texture and radial furrows were seen. Also, the dermatophyte test medium (DTM) at 25°C identiﬁed the growth of dermatophyte, as shown in Figure 2. The pathogen was identiﬁed as *Trichophyton tonsurans*.

**Figure 3:**
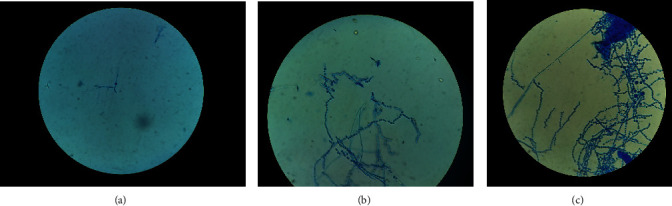
LPCB mount. (a) Curved septate hyphae as seen on the 5^th^ day. (b) (i) Abundant microconidia and few macroconidia. (ii) Intercalary and terminal chlamydospores. (c) Typical “bird on wire” pattern of microconidia.

Lacto Phenol Cotton Blue (LPCB) mount revealed curved septate hyphae and other features characteristic of *Trichophyton*, as shown in Figure 3.

Tissue sent for histopathology revealed skin ulceration with marked neutrophilic inﬁltrates, lymphocytes, and plasma cells in the dermis, along with few aggregates of epitheloid histiocytes and giant cells.

**Figure 4:**
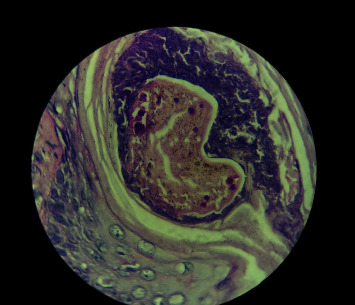
Histopathological features of the endothrix type of invasion.

Also, the typical presence of fungal spores inside the hair shaft was seen, as shown in Figure 4.

The isolate was conﬁrmed by MALDI-TOF as *Trichophyton tonsurans*.

**Figure 5:**
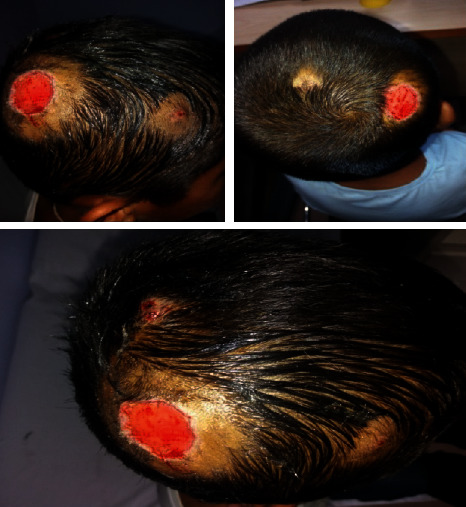
Follow-up at 1 week.

The patient received daily dressings for the next couple of days along with griseofulvin as systemic antifungal therapy for 6 weeks. A gradual and signiﬁcant clinical improvement was noted, as shown in Figure 5.

He was followed up for two years during which no recurrence was noted. However, the lesions healed with focal alopecia.

## 3. Discussion

Dermatophytes are the most common cause of fungal infections worldwide, infecting millions of individuals annually. Of these, tinea capitis tends to affect mostly the prepubertal children in crowded communities in a background of low socioeconomic status [3].

Kerion Celsi, a typical form of tinea capitis, may develop as a T-cell-mediated hypersensitivity reaction [4] or as an abundant host immune response to fungal infection [5]. The classical kerion consists of a painful, boggy, inﬂammatory mass associated with alopecia that may be solitary or multiple or, sometimes, even suppurated and covered with a thick crust. Although tinea capitis can affect individuals of any age, with cases reported as early as six days of life and as late as 70 years of age, a vast majority of patients seem to be in the prepubescent group consisting of children between 3 and 7 years of age [6].

Most of the *Trichophyton* species are geophilic, of which *T. tonsurans* is said to easily survive outside the host and is reported to be the only anthropophilic fungus that has been isolated in soil samples [7]. The transmission of *T. tonsurans* may occur directly (by infected or asymptomatic human carriers) or indirectly (fomites). The possible source of the infection in our patient was probably the contact of the scalp with soil during the fall, leading to dermal implantation of the infective agent. Investigations were sent after the lesion was excised, suspecting a bacterial origin. Many scalp and hair disorders may mimic the clinical presentation of kerion such as seborrheic dermatitis, bacterial folliculitis, psoriasis, trichotillomania, alopecia areata, and discoid lupus erythematosus. As a result, kerion is often underrecognized or confused with other lesions, leading to misdiagnosis and a subsequently delayed or inappropriate treatment [8]. This has been identified by a few studies [9, 10]. The burden of this disease has been estimated in 16 countries to date with a total of 21,073,423 cases, the majority of these being in sub-Saharan Africa and amongst school children. A recent review revealed a change in the last 1-2 decades and the spread of *Trichophyton tonsurans* as the dominant agent of tinea capitis, in the Americas, the UK, Europe, and Africa [11]. The burden of fungal infections in Nepal has long been underestimated with few diagnostic laboratories that are equipped with standard facilities to appropriately diagnose these conditions [12].

Once diagnosed, kerion can be treated with various systemic antifungal agents such as griseofulvin, terbinaﬁne, itraconazole, or ﬂuconazole. A 2008 meta-analysis had found griseofulvin to be an effective therapy for tinea capitis [13]. Also, a Cochrane review had stated that the efficacy of these medications is similar in the treatment of tinea capitis due to the *Trichophyton* species [14]. Therefore, following the traditional treatment of choice, our patient was also administered griseofulvin with a satisfactory outcome at 2-year follow-up.

## 4. Conclusions

1. High index of clinical suspicion is required to avoid misdiagnosis of kerion
2. Clinical ﬁndings, KOH mount, and culture reports complement each other in the diagnosis
3. Fungal culture is a necessary adjunct to direct microscopic examination for deﬁnitive identiﬁcation of etiological agents
4. The treatment can be more effective when antifungal therapy is based on the identiﬁcation of fungal isolates
